# Local Extinction in the Bird Assemblage in the Greater Beijing Area from 1877 to 2006

**DOI:** 10.1371/journal.pone.0039859

**Published:** 2012-06-29

**Authors:** Philippe Chouteau, Zhigang Jiang, Benjamin D. Bravery, Jing Cai, Zhongqiu Li, Miguel Pedrono, Olivier Pays

**Affiliations:** 1 Institute of Zoology, Chinese Academy of Sciences, Beijing, China; 2 Kexue Communications, Beijing, China; 3 Centre de Coopération Internationale en Recherche Agronomique pour le Développement, Montpellier, France; 4 Université d’Angers, Groupe Ecologie et Conservation, Angers, France; Hungarian Natural History Museum and Eotvos University, Hungary

## Abstract

Recent growth in industrialization and the modernization of agricultural activities, combined with human population growth, has greatly modified China’s natural environment, particularly in the vicinity of large cities. We compared avifauna checklists made between 1877 and 1938 with current checklists to determine the extent of local bird extinctions during the last century in the greater Beijing area. Our study shows that of the 411 bird species recorded from 1877–1938, 45 (10.9%) were no longer recorded from 2004–2006. Birds recorded as ‘rare’ in 1938 were more likely to have disappeared in subsequent years. Migrant status also influenced the probability of local bird extinction with winter migrants being the most affected class. Moreover, larger birds were more likely to have disappeared than smaller ones, potentially explained by differential ecological requirements and anthropogenic exploitation. Although our habitat descriptions and diet classification were not predictors of local bird extinction, the ecological processes driving local bird extinction are discussed in the light of historical changes that have impacted this region since the end of the 1930 s. Our results are of importance to the broader conservation of bird wildlife.

## Introduction

Under a global biodiversity crisis context [1], [2], [3], [4], the effect of global changes on biodiversity dynamics in China has received much attention [5], [6], [7], [8]. China is one of the most important countries for biodiversity conservation because it possesses important biodiversity hotspots and many endemic species [9]. However, with the increasing human population and transition from an agricultural to industrial economy since the Second World War, China now confronts major challenges related to the environment and biodiversity conservation [10], [11].

The majority of current agricultural land occupies former primary forests, grasslands and wetlands. Modification has resulted in the loss of biodiversity associated with the disappearance of natural habitats [12]. Freshwater habitat degradation due to human activities has been another factor causing biodiversity decline in China [13]. China has also experienced rapid urbanization since the 1980 s [14], with substantial effects on biodiversity caused by a reduction in natural habitat and an increase in the number of non-native species.

This study aims to demonstrate the impact of environmental changes on biodiversity at a regional scale, and over a long period of time, focusing on avifauna in the greater Beijing area. We study the local extinctions that occurred among the bird species assemblage in the Greater Beijing Area, due to changes that affected this area beginning in the 1930 s. The Greater Beijing Area comprises Beijing City, Tianjing City and Hebei Province and this region was known as Zhili province during the Qing Dynasty 1644−1911. The Greater Beijing Area is still a relatively well-defined biogeographic region [15]. Birds are considered good indicators of environmental change because they are easy to identify and their ecology and habitat requirements are well-known [16]. Bird checklists are a useful tool to indicate faunal change over time [17], [18], [19]. Here, we combine historical and modern records of birds and their habitats in order to track the fates of species observed between 1877 and 2008. We aimed to identify drivers of potential biodiversity decline by testing the effect of phenotypic (body size), ecological (habitat used, diet, migrant status) and population (abundance) factors on the probability of local bird extinction in the greater Beijing area.

We tested body size as a factor explaining local bird extinction, because there is a known negative relationship between bird abundance and body size [20]. Large individuals require more resources than smaller ones and so tend to occur at lower densities. Moreover, larger birds are more prone to extinction as they often have low fecundity (e.g. high age at maturity, low clutch size and long generation time), greater home ranges and low abundance, making them slower to respond to environmental threats. Additional factors include human exploitation of species of larger body sizes for food (e.g. pheasants, ducks) and the introduction of predators that disrupt the balance between fecundity and mortality [21]. They often need larger home ranges, forcing them to maintain low population densities, and making them sensitive to habitat fragmentation [22]. A rare bird species (e.g. one with a small population, restricted geographical range and low density) is more prone to extinction than an abundant species [23]. Abundance is linked to demographic characteristics of a species such as fecundity, rate of growth, generation time and longevity. Birds with low fecundity are often more prone to extinction because they take longer to recover if they are reduced to small population size and also if their mortality rate increases [21], due to human pressures, for example.

The Greater Beijing Area is also an important zone for bird migration in northeastern Asia because of its location between the sea and the Mongolian Desert [24]. Therefore, knowledge of migrant status is important for bird conservation. Resident birds are supposed to be more resilient to local extinction than passage migrant birds, because migrant birds may follow inflexible migration routes on which they face local threats such as the destruction of resting and feeding habitats used for refueling [25], [26]. In addition, hunting is also a threat at places for winter residents. Summer breeders are sensitive to habitat loss and fragmentation in breeding areas if they can no longer find suitable places for nesting [27]. They may also be sensitive to changes in climate that increase the frequency of extreme weather events and alter temperatures at different latitudes. The identification of wintering and breeding ranges as well as stopover sites is important for effective conservation of migratory birds [28]. Migratory birds may abandon destroyed stopover sites (or use them differently) and this may simulate an apparent local extinction [29].

Habitat destruction is one of the most important factors driving bird extinction (Bennett and Owens 2002). Some habitats like forest and wetlands are particularly susceptible to land conversion. In this study we wanted to focus on wetlands and forests because both these land types were heavily impacted by draining and pollution (wetlands, [30]) and clearing for agriculture (forests, [31]).

Last, we analyzed diet as a potential factor explaining local extinction, because a previous study showed that local bird extinction can be linked to diet [32].

In this study we did not investigate recent colonization of our studied area by bird species never previously recorded in the area, because the period of observation was too short (four years) to evaluate whether these recent observations corresponded to vagrancy or indicate extension of a species’ range [33]. The ecological processes driving local bird extinction are discussed in the light of the historical changes that impacted this region of China since the end of the 1930 s.

## Methods

### Study Area

**Figure 1:**
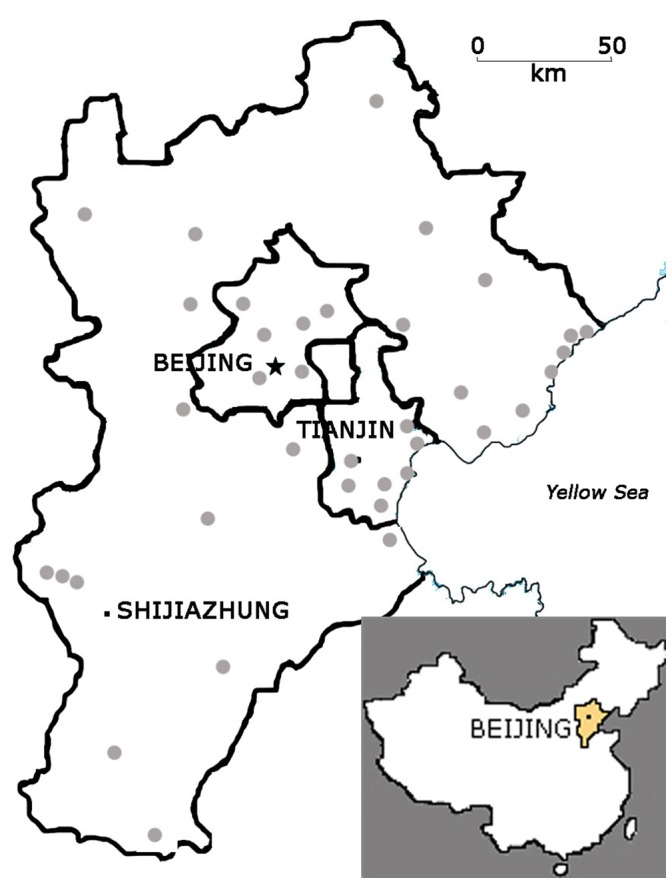
Map of the Greater Beijing Area. The Greater Beijing Area includes the Beijing and Tianjin Municipalities and Hebei Province. Grey spots indicate observation sites during the period 2004−2006.

The greater Beijing area is located in northern China (Figure 1) and covers 216,000 km^2^. The number of human inhabitants is around 100 million and has doubled since 1949.

### Past and Recent Avifauna Records

We focused on the Greater Beijing Area because of the great deal of historical information available regarding bird species in the region. Our aim was to make a checklist of birds recorded in the Greater Beijing Area from 1877 (when appropriate data were first collected) to 1938, a period in which the environment and avifauna changed very little. In 1937, war between China and Japan initiated profound and on-going changes to China’s environment that are still apparent today.

For the year 1877, we compiled a checklist using data published by Père Armand David, a French missionary who lived in China and studied natural history there from 1862–1874. The main source was the book ‘Les Oiseaux de la Chine’ (‘*The Birds of China*’ [34]), in which David recorded the places in China where he saw birds. We complemented this with a list of birds published in the ‘*Nouvelles Archives du Museum*’ [35]. A third source was an unpublished manuscript found at the French ‘Congregation of the Mission’ in Paris, to which David belonged, and that also gave a list of birds observed in the Greater Beijing area. The final checklist formulated from these three sources indicated that David recorded 397 species in the Greater Beijing Area in 1877.

For the period 1930–1938, we used the checklists obtained from ‘*Birds of the Hopei (Hebei) Province*’ [36] and ‘*Birds of Northeastern China*’ [37]. Shaw [36] studied birds for eight years and recorded 398 species. Wilder & Hubbard [37] compiled observations made from 1890–1937 and recorded 396 species. Different authors recorded different species in the same area, their studies having had different aims and probably also different methods to collect data. These differences led to some species being missed (e.g. some birds recorded by David were not recorded during the 1930 s, but were recorded again in the years after 2000), so we decided to merge David’s list and the 1930 s list to obtain a reliable unique list with 411 bird species observed in the Greater Beijing Area at any time from 1877–1938.

A list of birds recorded during from 2004–2007 was compiled from our personal observations in the Greater Beijing Area and from the ‘*China Bird Report*’ from 2004–2007 [38]. Around 60 people conducted observations at different spots across the study area (Figure 1). To belong to that 2007 list, birds had to be recorded in two or more years and by two or more different observers, in order to reduce misidentifications. This new list included 383 species that met the criteria and were recorded during the four years of observation (including ten species that were never recorded in the region previously and seven species split from other species after 1938).

Bird nomenclature has changed during the last century. Some of the species identified by David, Shaw and Wilder and Hubbard are now reduced to subspecies level, and others have been invalidated. To unify the nomenclature across different lists we followed the nomenclature of Brazil [39], complemented, for the orders and families, by the phylogeny provided by Paterson [40], and Gill and Donsker [41]. To reduce doubt in bird identification we used plates provided by David [34] and drawings from Shaw [36] and Wilder & Hubbard [37] to identify species. The fact that many observers, although of different skill, made current observations in the area should have eliminated bias due to sampling effort and species detection, which may have led to an underestimation of the number of species in an area [17].

We considered bird species currently locally extinct from the Greater Beijing Area if they were recorded by David, Shaw or Wilder and Hubbard (i.e. during the first period), but were not recorded 2004–2007.

### Index of Ecological Characteristics and Body Size

We obtained no quantitative data or detailed information on species abundance from the different authors. However, Wilder & Hubbard [37] gave relatively precise qualitative data (‘common’, ‘many individuals’, or ‘rare’) for almost all bird species. Although it may be difficult to evaluate trends in abundance with qualitative data, previous studies have used such information to assess bird abundance over long periods of time when no other information is available [42]. The level of information provided in the checklist made by Wilder & Hubbard [37] was sufficient for us to assign each species to a category and we also checked, if available, with the abundance data provided by Shaw [36]. We pooled the categories ‘common’ and ‘many individuals’, and ‘few individuals’ and ‘rare’ birds. Birds (28 species) in the list for 1877–1938 and for which we have no qualitative abundance data were assigned to a separate ‘undetermined’ class. Therefore, abundance for each bird species was categorized into one of three classes: common, rare or undetermined.

We determined habitat, diet, resident or migrant status and body size using features described in MacKinnon & Phillipps [43] and Brazil [39], enhanced with personal observations. Categories were chosen according to previous studies into local bird extinction that fit the statistical analysis we performed.

We used the following categories to characterize habitat: forest (habitat with a high tree density, with a dense umbrella-like canopy, 20−30 m height, and with little ground cover vegetation), woodland (habitat with tree density decreasing compared to forest, with more opened canopy, canopy around 20 m high, and with a ground cover vegetation more important compared to forest), scrub (habitat with woody plants 2–8 m tall with many stems), farmland (area dominated by agricultural landscapes), open area (landscape dominated by natural grassland), water (including open water as river), bank (river bank and seashore) and wetland (marshes and swamps).

We defined the following diets: carnivore (including birds feeding on fish), granivore, herbivore, insectivore (birds feeding on insects), frugivore, insectivore-granivore, insectivore-frugivore and omnivore (bird eating insects, seeds and fruits),

We defined the following migratory statuses: resident (recorded all year), summer breeder (recorded only during summer), passage migrant (recorded only during spring or autumn), and winter visitor (recorded only during winter). For the few species that exhibit mixed migration status we retained the dominating ones as defined in Brazil [39].

Body-size measurements were assessed according to MacKinnon & Phillipps [43] and Brazil [39]. When body length differed between the sexes for a given species, an average of male and female measurements was used.

### Data Analysis

We tested the effect of abundance, body size, diet, habitat type and migrant status on the probability of local apparent extinction in birds, using generalized linear mixed-effects models (GLMM) and Laplace approximation (Link function  =  Logit, Binomial) [44]. To improve goodness of fit in the relationships between those variables, body size was log-transformed. In the models, log-transformed body size was a continuous factor and abundance, diet, habitat, and migrant status were categorical factors. Phylogenetic effects may underlie similarities between species and to limit bias due to phylogenetic clustering we included Genus within Family within Order as three nested random factors. To reduce the large number of degrees of freedom in our statistical procedures triggered by the large number of continuous and categorical variables, we included only those two-way interactions that were interpretable in terms of mechanisms influencing the probability of local extinction (Table 1). Multicollinearity was limited in all models and statistical inferences were valid, as variance inflation factors were consistently less than 10 [45].

**Table 1:** Akaike’s criterion (AIC) and the corrected criterion (AICc) for each candidate model explaining the variation in probability of local apparent extinction of birds in China using GLMM Laplace procedure (Link  =  Logit, Binomial) including log-transformed body size as a continuous, diet (*carnivore*, frugivore, granivore, herbivore, insectivore, insectivore-frugivore, granivore-insectivore, omnivore), habitat (*bank*, farmland, forest, marsh, open area, scrub, water, woodland), and migrant status (*migrant*, resident, summer visitor, winter visitor) as categorical factors, abundance (*common*, rare, undetermined) (with italicized class used as reference), and Genus within Family within Order as three nested random factors.

ID Model	Model description	LogLik	K	AIC	AICc	ΔAICc	Wi
Null model		−138.04	4	282.10	282.20	51.45	0.00
1	Log(Body size)	−133.81	5	275.60	275.75	45.00	0.00
2	Diet	−136.54	11	293.10	293.76	63.01	0.00
3	Habitat	−133.58	12	289.20	289.98	59.23	0.00
4	Migrant Status	−131.30	7	274.60	274.88	44.13	0.00
5	Abundance	−116.10	6	242.20	242.41	11.66	0.00
6	Log(Body size) + Diet	−131.63	12	285.30	286.08	55.33	0.00
6i	Log(Body size) + Diet + Log(Body size)*Diet	−124.74	19	285.50	287.44	56.69	0.00
7	Log(Body size) + Habitat	−128.98	13	282.00	282.92	52.17	0.00
8	Log(Body size) + Migrant Status	−125.67	8	265.30	265.66	34.91	0.00
8i	Log(Body size) + Migrant Status + Log(Body size)*Migrant Status	−125.35	11	270.70	271.36	40.61	0.00
9	Log(Body size) + Abundance	−113.45	7	238.90	239.18	8.43	0.01
9i	Log(Body size) + Abundance + Log(Body size)*Abundance	−112.63	9	241.30	241.75	11.00	0.00
10	Diet + Habitat	−131.53	19	299.10	301.04	70.29	0.00
11	Diet + Migrant Status	−129.58	14	285.20	286.26	55.51	0.00
12	Diet + Abundance	−113.65	13	251.30	252.22	21.47	0.00
13	Habitat + Migrant Status	−127.29	15	282.60	283.82	53.07	0.00
14	Habitat + Abundance	−111.30	14	248.60	249.66	18.91	0.00
15	Migrant Status + Abundance	−110.39	9	236.80	237.25	6.50	0.03
15i	Migrant Status + Abundance + Migrant Status*Abundance	−107.62	15	243.20	244.42	13.67	0.00
16	Log(Body size) + Diet + Habitat	−127.07	20	292.10	294.25	63.50	0.00
17	Log(Body size) + Diet + Migrant Status	−124.68	15	277.40	278.62	47.87	0.00
18	Log(Body size) + Diet + Abundance	−110.99	14	248.00	249.06	18.31	0.00
19	Log(Body size) + Habitat + Migrant Status	−121.79	16	273.60	274.98	44.23	0.00
20	Log(Body size) + Habitat + Abundance	−109.12	15	246.20	247.42	16.67	0.00
**21**	**Log(Body size) + Migrant Status + Abundance**	**−106.12**	**10**	**230.20**	**230.75**	**0.00**	**0.72**
21i	Log(Body size) + Migrant Status + Abundance + Log(Body size)*Migrant Status	−105.92	13	235.80	236.72	5.97	0.04
21ii	Log(Body size) + Migrant Status + Abundance + Log(Body size)*Abundance	−105.35	12	232.70	233.48	2.73	0.18
21iii	Log(Body size) + Migrant Status + Abundance + Migrant Status*Abundance	−103.45	16	236.90	238.28	7.53	0.02
22	Diet + Habitat + Migrant Status	−124.90	22	291.80	294.41	63.66	0.00
23	Diet + Habitat + Abundance	−108.77	21	257.50	259.88	29.13	0.00
24	Diet + Migrant Status + Abundance	−107.77	16	245.50	246.88	16.13	0.00
25	Habitat + Migrant Status + Abundance	−106.22	17	244.40	245.96	15.21	0.00
26	Log(Body size) + Diet + Habitat + Migrant Status	−120.75	23	285.50	288.35	57.60	0.00
27	Log(Body size) + Diet + Habitat + Abundance +	−107.06	22	256.10	258.71	27.96	0.00
28	Log(Body size) + Diet + Migrant Status + Abundance	−104.84	17	241.70	243.26	12.51	0.00
29	Diet + Habitat + Migrant Status + Abundance	−103.80	24	253.60	256.71	25.96	0.00
30	Log(Body size) + Diet + Habitat + Migrant Status + Abundance	−101.94	25	251.90	255.28	24.53	0.00

LogLik is the loglikelihood; k is the number of estimated parameters. The best model with the lowest AICc is in bold. ΔAICc is the difference between that model’s AICc and the best one; ω_i_ is the weight of the model indicating the probability that a given model is the best among the model candidates. The null model included only the Family random factor without the addition of fixed effects.

We derived all possible candidate models (Table 1). Akaike’s criterion (AIC) and the corrected criterion (AICc) for each candidate model were calculated including the null model [46]. The best model had the lowest AICc and the highest weight (ω_i_) indicating the probability of being the best among all model candidates. As AIC does not give P-values and to test for significant effect of factors in the best model, using χ^2^ tests we compared the deviance between the best and a candidate model without the studied factor for which we wanted to assess the P-value. If the deviance between the two models was significant, the studied factor had a significant effect on the probability of local extinction. Finally, because of the ‘undetermined’ class for abundance of several species, the effect of abundance on the probability of local bird extinction was considered significant only when (1) the best model (with the lowest AICc) included abundance, and (2) the deviance between the two models and (3) the difference of local extinction between ‘rare’ and ‘common’ species was significant. All statistical analyses were performed using R 2.10.1 [47].

## Results

**Figure 2:**
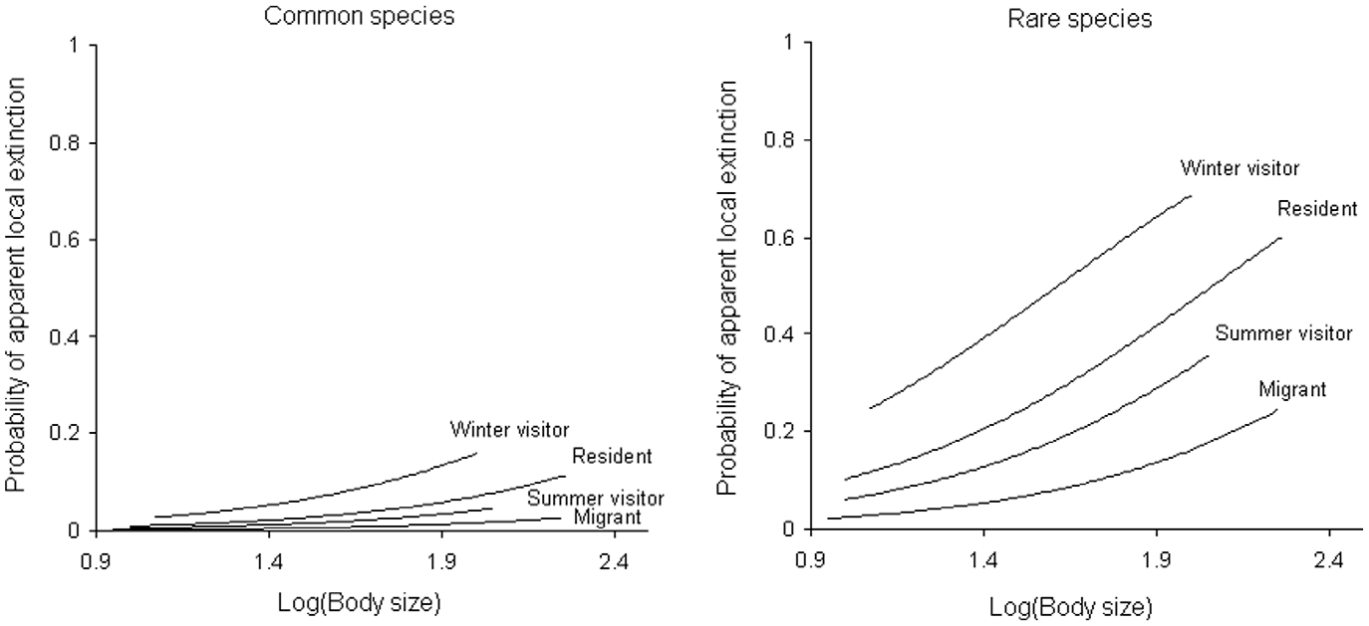
Relationship between the log-transformed body size and the probability of apparent local extinction. The relationship is explained for each category of abundance, for ‘common’ and ‘rare’ bird species recorded in the study area (see Table 3 for statistical details).

The model that best explained variation in the probability of local extinction of bird species included body size (log-transformed), migrant status and abundance (Table 1). Deviances between models 21 (i.e. the best model) and 15 (model without log-transformed body size), 21 and 9 (model without migrant status) and models 21 and 8 (model without abundance) show that body size, migrant status and abundance significantly affected the probability of local bird extinction (Table 2). According to the coefficient derived for each variable (Table 3), the probability of local bird extinction increased with log-transformed body size. Rare species of birds were more likely to experience local extinction than common species. Secondly, controlling for body size and abundance, differences were found in the probability of local extinction in relation to a bird’s migrant status (Figure 2). Winter visitors were more inclined to undergo local extinction whereas migrants (visitors both in spring and autumn) were less affected by local extinction and residents occupied an intermediate position. Finally, bird habitat and diet contributed very little to explaining variation in the probability of local extinction (Table 1).

**Table 2:** Analysis of deviance between the best and candidate model, testing for significance of the effect of Log(Body Size), Migrant Status and Abundance on the probability of apparent local extinction of bird species in the studied region of China. Each model is fully described in Table 1.

Models	Tested factor	χ^2^	df	P
21 versus 15	Log(Body Size)	8.54	1	0.003
21 versus 9	Migrant Status	14.66	3	0.002
21 versus 8	Abundance	39.11	2	<0.001

**Table 3:** Estimate (± SE) of factors that explained variation in the probability of apparent local extinction of bird species in the studied region of China (using a GLMM Laplace procedure (Link  =  Logit, Binomial) with Genus within Family within Order as three nested random factors.

Factors	Estimate	SE	P
(Intercept)	−7.957	1.252	<0.001
Log(Body Size)	1.977	0.649	0.002
Migrant Status (when *migrant* is the reference)			
Resident	1.520	0.504	0.003
Summer Visitor	0.943	0.477	0.048
Winter Visitor	2.449	0.646	<0.001
Abundance (when *common* is the reference)			
Rare	3.696	0.739	<0.001
Unknown	2.400	0.637	<0.001

### Changes in Bird Assemblage

The database included 411 species. Local extinction rates among bird families in which extinctions were recorded are shown in Table 4.

**Table 4:** Percentage of local extinctions in some bird families in the Greater Beijing Area during the period 1938−2007 (only families in which extinctions were recorded are indicated).

Families	Number of species	Species extinct	%
Phasianidae	11	5	45.4
Anatidae	34	3	8.8
Ciconiidae	3	1	33.0
Threskiornithidae	4	3	75.0
Pelecanidae	2	1	50.0
Phalacrocoracidae	3	2	66.6
Accipitridae	28	6	21.4
Jacanidae	1	1	100.0
Scolopacidae	37	1	2.7
Columbidae	6	2	33.0
Strigidae	9	1	11.1
Picidae	8	1	12.5
Laniidae	5	1	20.0
Dicruridae	3	1	33.3
Corvidae	18	3	16.7
Paridae	7	1	14.3
Acrocephalidae	6	1	16.7
Locustellidae	5	1	20
Muscicapidae	23	4	17.4
Passeridae	6	1	16.7
Fringillidae	17	4	23.5
Emberizidae	17	1	5.9

Twenty-seven bird species [e.g.: Blood Pheasant (*Ithaginis cruentus*); Black Grouse (*Tetrao tetrix*); Steller’s Sea Eagle (*Haliaeetus pelagicus*)] were no longer observed in the 1930 s by Wilder & Hubbard [37], and 25 other species (e.g.: Pallas’s Fish Eagle (*Haliaeetus leucoryphus*); Eurasian Eagle Owl (*Bubo bubo*); Grey-headed Woodpecker (*Picus canus*)] were recorded with less than two individuals. Shaw [36] or Wilder & Hubbard [37] recorded 19 species not recorded by David [e.g.: Spotted Capercaillie (*Tetrao parvirostris*); Japanese Sparrowhawk (*Accipiter gularis*); Eurasian Pygmy Owl (*Glaucidium passerinum*)]. Twelve species that were recorded as very rare before 1938 (with only one or two records made by Wilder & Hubbard) were often recorded (>50 records) in 2004–7 [e.g.: Little Egret (*Egretta garzetta*); Red-crowned Crane (*Grus japonensis*); Light-vented Bulbul (*Pycnonotus sinensis*)]. Forty-five species encountered before 1938 were no longer recorded during recent surveys (11.5% of species locally extinct, compared to 1877–1938) with 12 species already locally extinct before the 1930 s and six which had been recorded by Shaw or Wilder & Hubbard but not by David; 29 of these species (64.6%) were recorded as rare by Wilder & Hubbard. Only three species now locally extinct (6.3%) were recorded as common in the 1930 s: Pelagic Cormorant (*Phalacrocorax pelagicus*), Collared Crow (*Corvus pectoralis*) and Streaked Reed Warbler (*Acrocephalus sorgophilus*) a now-rare endemic of Eastern Asia, for which the Greater Beijing Area encompasses the biggest part of the breeding area [39]. Among other now locally extinct bird species, Japanese Cormorant (*Phalacrocorax capillatus*) is also an endemic to East Asia [39]. Our complete database is presented in Appendix 1.

## Discussion

### Body Size and Abundance

It appears that local extinction among species in the Greater Beijing Area occurred mainly among large birds already rare in the 1930 s. Our results accord with reported links between body size and local bird extinctions [21] as large birds are more prone to extinction due to human pressure. Among the large birds extinct in our study, pheasants, waterbirds and raptors were directly persecuted by hunting or endured indirect threats as human pressure increased [48].

Hunting is a cause which accounts for biodiversity decline and may have a severe impact on large birds in China [49]. For example, the Daurian Partridge (*Perdix dauurica*) was heavily impacted by hunting in our focal area: this species was considered very abundant in 1877 [34] but was only observed 23 times during the period 2004–2007. Other birds were also hunted for use in traditional Chinese medicine (e.g. eagles) or to collect feathers for clothes and ornaments (e.g. kingfishers). Hunting of birds was more common after 1937 because of the war between China and Japan resulted in a reduction in food supply [50] and because guerilla fighters fed on wild animals. The end of the war was followed by a great famine in China in 1946. During the Chinese civil war (1945–1949), fighting occurred in the area, until the communist army took over Beijing in February 1949. Heavy hunting over such a long period of time depleted populations of game birds, and was one of the causes of local extinction of pheasants in the study area. Previous studies have reported that hunting triggered the local extinction of pheasants, such as the Blood Pheasant (*Ithaginis cruentus*), Reeves’s Pheasant (*Syrmaticus reevesii*), Hazel Grouse (*Tetrastes bonasia*), Black Grouse (*Tetrastes tetrix*) and Black-billed Capercaillie (*Tetrao parvirostris*) in the Greater Beijing Area [48].

Hunting with guns is now severely restricted [49], [51]. However, other forms of hunting are probably still widespread (e.g. the use of poison baits and nets) as well as the illegal destruction of nests and egg collection, e.g. Brown Eared Pheasant (*Crossoptilon manchuricum*, [48]). These threats have an important impact on forest-dwelling birds, where they can be easily practiced by poachers, but they are probably less efficient for waterbirds, such as ducks, due to the difficulties of poaching in open areas in China [52].

Another explanation for local waterbird extinction is food shortages due to the disappearance of small aquatic animals affected by the excessive use of chemicals and habitat destruction in China [53]. Crested Ibis (*Nipponia nippon*) may have disappeared from the area because of such food shortages, rather than hunting, because David reported its flesh was unpalatable. The Ibis fed on crabs, frogs, small fish, river snails, other mollusks and beetles [48]. Disappearance of these animals, in addition to modification of its habitat (destruction of tall nesting trees, modification of river banks due to urbanization) contributed to its local extinction [48]. Overfishing may also explain the local extinction of big waterbirds, such as pelicans, storks, spoonbills, ibises and cormorants, by depleting the food these birds usually feed on [48]. Indirect human pressure such as modification of the surroundings of wetlands where waterbirds forage can also drive some large species to local extinction. For example, the Pheasant-tailed Jacana (*Hydrophasianus chirurgus*) lived in ponds with extensive surface vegetation, on which it forages. However, this kind of wetland disappeared in the Greater Beijing Area because of the conversion of wetlands for agriculture, and the lack of surface vegetation in the water reservoirs constructed as source of human drink water.

Large raptors (Accipitridae) also underwent serious decline. In the Greater Beijing Area, they may suffer from direct persecution (Eastern Imperial Eagle, *Aquila heliaca*), a reduction in habitat (Pallas’s Fish Eagle, *Haliaeetus leucoryphus*), food depletion due to overfishing (Steller’s Fish Eagle, *Haliaeetus pelagicus*), changes in agricultural practice (Lammergeier, *Gypaetus barbatus*) and the over-use of pesticides (Bonelli’s Eagle, *Aquila fasciata*) [48]. Moreover, raptor density depends upon specific feeding habits, and habitat changes may have a direct effect on prey abundance and community structure [54]. Indeed, in our study area, many open areas in which some raptors previously captured prey have been converted to agriculture or urbanized [55].

### Migrant Status

The Greater Beijing Area is also an important zone for bird migration in North-East Asia, because of its location between the sea and the Mongolian Desert, inducing migrating birds to favor this route [24].

Winter-visitor birds (i.e. migrants wintering in the study area) were more prone to local extinction than others. All but one locally extinct winter migrant were small birds and hunting cannot be the main cause of extinction in the study area. However, we can hypothesize that hunting was probably more intensive during periods of food shortage, and probably even these small species were severely impacted. They may have also been impacted by the pet trade that constitutes of mainly small birds. The observed trend may also be due to global climate change taking place across northern China: temperatures have increased during the last century in the study area, with a warming period starting in the 1920 s [56], [57]. Indeed, birds that previously spent the summer in Northern Siberia or Central Asia and migrated to the Greater Beijing Area during winter in the 1877–1938 period would now find more suitable conditions in their breeding area during winter, and no longer need to migrate to the study area, indicating a range shift. Some species currently have their wintering range outside the Greater Beijing Area, e.g. Great Grey Shrike (*Lanius excubitor*), Arctic Redpoll (*Carduelis hornemanni*), Asian Rosy Finch (*Leucosticte arctoa*), Przewalski’s Redstart (*Phoenicurus alaschanicus*) and Güldenstädt’s Redstart (*Phoenicurus erythrogaster*). None of these species are currently threatened by extinction on a global scale [48].

Global change may not significantly impact passage migrant birds because the large size of the area still allows them to maintain suitable stopovers [28]. However, a decline of passage migrant bird populations could occur outside the Greater Beijing Area and then negatively impact the area’s migrant populations. For example, the Snow Goose (*Anser caerulescens*), once common in East Asia [39], suffered a severe decline in Northern Siberia where this species breeds during summer [42] and this event reduced the number of migrant individuals passing over the Greater Beijing Area during migration.

Breeding summer birds and resident birds were slightly affected in the Greater Beijing Area. It is possible there are currently fewer suitable nesting places in the area, explaining the slight decrease in abundance revealed by our study. For example, native forests have been replaced by tree plantations that offer fewer possibilities for nesting. The Black Woodpecker (*Dryocopus martius*) and the Eurasian Pygmy Owl (*Glaucidium passerinum*) became locally extinct, although previously they were commonly recorded near Beijing [34]. These two species inhabit mature forests with old trees for nesting [39], and young plantations are not suitable for these birds.

Finally, an underestimation of birds during winter may explain the observed trend. In our database, more bird observations were collected during summer than winter, suggesting that fewer birds were observed during winter in the Greater Beijing Area. We collected 864 bird observations (from a total of 4393) made from November 1 to March 15 (corresponding to the coldest period in north China), or 19.7% of the total observations for more than one third of the year. This result suggests birds are rare during winter in the study area. An alternative explanation could be observers may be reluctant to go birdwatching during the cold winter: birds are less observed, and this underestimates the number of individuals and species occurring in the area. However, we have no data on sampling effort (e.g. number of hours spent watching birds) so we cannot distinguish these two explanations.

### Habitat Change in the Greater Beijing Area

We did not find a significant impact of habitat on local extinction. This could be due to the large size of the study area, where some tracks of relatively undisturbed habitat still exist, and in which birds are encountered. A study on a much smaller area (e.g. 1,000 km^2^) would have surely produced different results, because there are probably spatial patterns in bird abundance according to habitat distribution in the study area. We do not know exactly where David, Shaw and Wilder & Hubbard made their observations and we lack reliable maps of the recent extent of habitat during the 20^th^ century.

However, the hypothesis that habitat destruction had a significant effect on local extinction is plausible because native habitats in the Greater Beijing Area were converted during the 20^th^ century for agriculture and urbanization. For example, in an area situated in Yanshan Mountain (17,371 km^2^), in northern Beijing, the cultivated land area was 3,670 km^2^ (21% of the area) in 1958 [58]. By 1979, the area used for cultivation increased to 7,470 km^2^ (43%), but from 1979, the land cultivated decreased to 5,930 km^2^ (34%) in 2001 [58]. This trend was observed in the Greater Beijing Area [55] and it was not due to abandoned cultivated land or afforestation, but due to urban expansion caused by economic development and population growth over the past two decades [59], [60].

Although forest destruction was probably intense before David performed his observations, some places were still covered with undisturbed forests in 1877–1938. The forest near Chengde, Hebei was a large game reserve for Chinese emperors during the Qing Dynasty, and stayed relatively undisturbed until 1920. However, disappearance of forest during the 20^th^ century in China was partly counteracted by the reforestation policy commencing in the 1950 s, and has been followed intensively [31]. The Greater Beijing Area was covered by 162,390 km^2^ (75%) of forest in Pre-Historic times [31]. Around 1948, total forest cover was between 1,260–6,350 km^2^ (0.8–3.9% of original forest cover) but forests have been planted and the forested area was 22,880 km^2^ or 11% of the region in 1988 [31]. In northern Hebei, forested areas increased by more than 200% from 1960 to 1987 [61]. Even if plantations are not as ecologically efficient as native forests, they are still useful for maintaining some resilient forest bird species, especially if those plantations are near primary forest. Studies in Japan [62] show that mixed plantations (pine and hardwood trees) maintain a higher number of bird species than pure coniferous plantations, suggesting that the mixing of broad-leaved trees in coniferous plantations is an effective way to increase bird diversity in plantations. This trend could also be true in China.

### Implications for Conservation

The Streaked Reed Warbler (*Acrocephalus sorgophilus*) is a narrow-restricted summer breeding bird in the Greater Beijing Area and in Liaoning Province [39]. Its disappearance from the area is probably due to the lack of reed beds where it breeds during summer [39]. Its conservation requires the conservation and restoration of remaining ponds.

Although an effect of habitat degradation was not revealed by our study, efforts to restore forest and to increase tree plantations around Beijing should be maintained at a high level, in order to protect Beijing from sandstorms [63], maintain the function of the forest ecosystem in water management [64] and connect different parcels of forest, that may be useful for the survival of some forest birds (e.g. the Brown Eared Pheasant) [48]. However, these plantations usually have low tree diversity and are sometimes monospecific [65]). We suggest that effort is needed to diversify tree species used for plantations in China and to use native tree species for reforestation [66]. In addition, plantations should be carefully managed, because more than 50% of the plantations created in China since 1949 have been degraded or have since disappeared [67].

### Checklist Considerations and Caveats

The fact some bird species were not longer recorded in recent surveys does not mean they will not be recorded again in the future. Because of the large size of the study area, and if the study was longer than four years, allowing to get more observations, some birds currently recorded as extinct could be observed again in the future, and rare birds could also be observed more often, especially because there are more and more birders operating in this area with better equipment and more facilities to explore the most remote areas. Increased sampling effort has recently provided more information about bird status, including observations of species never before recorded. For example, the Cinereous Vulture (*Aegypius monachus*), observed by David but considered extinct in the 1930 s was recently observed in the area. On the other hand, the Rock Dove (*Columba livia*), is believed to be locally extinct according to our study (although common in the 1930 s), but Brazil [39] pointed out that this species is often confused with domestic pigeons. A better survey could reveal this species is still in the area.

Information on historical bird repartition at other localities in China is scant and so we were unable to compare the degree of local extinctions across different places in China. It would be interesting to compare our results to other regions when these checklists become available.

Our work shows that checklists could also be useful for studying recent colonization of the area by new bird species, as a consequence of global climate change. However, our data did not allow us to do this because of only four years of observations. The relatively low numbers of individuals of the new species observed and the lack of breeding observations did not allow us to determine whether these species were vagrants or signified an enduring colonization of the area.
